# Proceedings of Terrell Medical Society

**Published:** 1895-03

**Authors:** James Orr

**Affiliations:** Secretary; Terrell, Texas


### Society Notes.

### Proceedings of Terrell Medical Society.

TERRELL, TEXAS, January 7, 1895.

The regular monthly meeting of Terrell Medical Society took place to-day, with a good attendance present. Dr. B. F. Church, President, presided, and Dr. James Orr, Secretary.

After routine business, Dr. Orr, for Committee on New Remedies, reported on the use of guiacol in sore throats, he having used it in twelve cases of acute tonsilitis, in three of which pharynx and pillars of fauces were affected, without a single failure, all the cases being promptly relieved. His method of applying the remedy is by means of a small mop, or camel's hair brush, by which it is applied, pure, to the affected parts, being sure to reach all the diseased surface. This treatment is repeated every three hours, until subsidence of the acute symptoms, not more than three applications usually being required.

Drs. Anthony and Monday have used guiacol to reduce the temperature after the method of Da Costa. They cleanse a small place on the epigastrium and rub ten drops of pure guiacol over it, then cover with paper and a few folds of cotton cloth. In a few minutes diaphoresis takes place, and temperature rapidly falls to normal.

Dr. Dumas reported an interesting case of puerperal septicæmia, in which he removed a number of disintegrating blood clots from womb four days after delivery.

Dr. Mathews reported two cases, in same family, simulating scarlatina so closely his inability to trace the origin of the infection alone prevented his announcing a diagnosis as such. The Society asked that this family be watched, to note any further development of the disease, in order to take precautions against its spreading if more cases develop and it should prove to be veritable scarlatina.

Dr. Orr contributed a paper on "Physicians who have died in Terrell," which was a brief resume of professional character and services of eleven physicians who have died here since 1879. In this a high compliment was paid Drs. F. D. Hallonquist, J. T. Webb, John Inabnit and H. L. Parsons. The paper was ordered printed.

Drs. Neeley and Dumas were appointed contributors for the next meeting, which takes place Monday, February 4, 1895.

Terrell Medical Society has been in existence since 1881, and is very prosperous, having a good membership of earnest workers. Its meetings are held the first Monday in each month, at 2:30 p. m., and are always full of interest.

JAMES ORR, Secretary.

